# Thirst in Patients With Advanced Chronic Heart Failure Waiting for a Heart Transplantation: A Mixed Methods Study of Patients' and Nurses' Experiences

**DOI:** 10.1111/nicc.70686

**Published:** 2026-10-07

**Authors:** Franziska Wefer, Lars Krüger, Stella Calo, Ralph Möhler, Martin Nikolaus Dichter, Andrea Mühring, Jan Gummert, Sascha Köpke

**Affiliations:** ^1^ Care Development, Care Directorate Ruhr University Bochum, Heart and Diabetes Center NRW Bad Oeynhausen Germany; ^2^ Faculty of Medicine & University Hospital Cologne Institute of Nursing Science, University of Cologne Cologne Germany; ^3^ Project and Knowledge Management/Care Development Intensive Care, Care Directorate Ruhr University Bochum, Heart and Diabetes Center NRW Bad Oeynhausen Germany; ^4^ Institute for Health Services Research and Health Economics Centre for Health and Society, Medical Faculty and University Hospital Düsseldorf, Heinrich Heine University Düsseldorf; ^5^ Center for Heart Failure, Care Directorate Ruhr University Bochum, Heart and Diabetes Center NRW Bad Oeynhausen Germany; ^6^ Department of Thoracic and Cardiovascular Surgery Ruhr University Bochum, Heart and Diabetes Center NRW Bad Oeynhausen Germany

**Keywords:** counselling, heart failure, mixed methods, nursing assessment, thirst

## Abstract

**Background:**

Hospitalised patients with advanced chronic heart failure (CHF) waiting for transplantation may experience increased thirst due to their critical illness and therapy‐related requirements. The perspective of patients and nurses is important for developing a nurse‐based counselling intervention to reduce thirst.

**Aims:**

To identify patients' and nurses' experiences and perceptions of thirst, as well as their information needs and preferences on information dissemination.

**Study Design:**

This convergent mixed methods study used semi‐structured interviews with patients, focus groups and a survey with nurses in Germany. We analysed the qualitative data using content analysis and the quantitative data descriptively. We compared the results of both methodological approaches and summarized the data as meta‐inferences.

**Results & Findings:**

We conducted 10 interviews with patients on intensive care units, intermediate care units and general wards during their waiting time for transplantation, two focus groups with, respectively, five nurses and a survey with 59 nurses caring for these patients. Analyses resulted in five common categories: experiences of thirst, factors that influence thirst, assessment of thirst, dealing with thirst and information about thirst. Patients' experiences with thirst differ markedly, ranging from mild to severe thirst, and they use various strategies to deal with thirst. Nurses consider thirst relevant for patients and nursing care. Strategies used in practice for assessment of thirst are very heterogeneous. In the survey, less than half of the nurses claimed to regularly assess patients' thirst. Nurses and patients wish to receive individual information and counselling.

**Conclusions:**

Thirst in hospitalised patients with advanced CHF waiting for transplantation can be severe and is experienced differently. Standardised procedures for assessing and managing thirst on the ward seem essential to address patients' needs and preferences.

**Relevance to Clinical Practice:**

Patients and nurses express a need for professional counselling on managing thirst in clinical practice.

## Impact Statements

- What is known about the topic
  ○ Thirst is often described in patients with CHF.
  ○ Different interventions such as using ice cubes, chewing gum or cold‐water spray were studied in the context of thirst in CHF.
- What this paper adds
  ○ Both patients and nurses demonstrate a need for professional advice regarding thirst in the context of advanced CHF.
  ○ Patients perceived their thirst individually and use specific personal strategies to alleviate it.
  ○ Nurses described high relevance for treatment of thirst in nursing care.

## 1 Introduction

Around 64 million people worldwide suffer from chronic heart failure (CHF), with an estimated prevalence of 3.9% in Germany in 2017 [1]. Patients with CHF experience various symptoms and a severely reduced quality of life [2]. For advanced CHF, ventricular assist devices (VAD) can serve as a bridge to transplantation, with heart transplantation being the final treatment option [3, 4]. While awaiting transplantation, patients often have a long inpatient stay in intermediate care (IMC) or intensive care units (ICU) and receive complex medical therapy and therapeutic restrictions, such as fluid restriction [5, 6]. Many also report increased thirst, the extent of which is influenced by the severity of CHF [7]. Thapa et al. [8] and Gong et al. [9] found moderate to severe thirst distress in up to 73% of CHF patients. Thirst also occurs during critical phases of illness, for example, on ICU [10].

### 1.1 Background

There is little evidence on interventions to reduce thirst in patients with advanced CHF [7, 11]. A recent systematic review summarised non‐pharmacological strategies for patients with CHF and those undergoing haemodialysis, including chewing gum, sucking frozen strawberries or ice cubes, acupressure and psychological interventions, but most studies were heterogeneous and of limited methodological quality [11]. Also, weak evidence exists for the use of mint or mume spray [7], cold water spray [12] and symptom management programs [13]. Two cross‐sectional studies showed that most patients drink fluids when thirsty, with few using specific interventions such as ice cubes [8, 14]. Another small study found that 95% of healthcare professionals recommended most frequently ice cubes [15]. In ICU populations, nursing strategies such as ice cold‐water spray, menthol water spray or the use of cold swabs have been reported [10, 16, 17, 18].

Given the weak evidence, the importance of patient preferences and cultural differences, evidence‐based counselling by nurses is warranted [19]. In order to develop such complex interventions, the perspectives of both patients and nurses must be considered [20].

### 1.2 Aims

Our aims are to identify patients' and nurses' experiences and perceptions of thirst as well as their information needs and how this information should be disseminated. This mixed methods study forms part of a larger project to develop and evaluate a nurse‐based counselling intervention to promote self‐care skills for managing thirst in hospitalised patients with advanced CHF awaiting transplantation. The results will inform the design of the intervention.

## 2 Methods

### 2.1 Study Design

Based on the Medical Research Council framework for developing and evaluating complex interventions [20], we conducted a convergent mixed methods study. We conducted guided interviews with patients on hospital wards waiting for a heart transplantation as well as focus groups (FG) and a survey with nurses experienced in the field. Study protocol was published elsewhere [19]. Our reporting complies with the criteria of Good Reporting of A Mixed Methods Study (GRAMMS) (Table S1) [21].

### 2.2 Recruitment and Sample

We recruited patients for the interviews and nurses for FG at cardiac surgery units and wards (ICU, IMC and general ward) in two German university hospitals. For the survey, we invited nurses from Germany who work in the care of patients with advanced CHF waiting for heart transplantation for participation.

#### 2.2.1 Interviews With Patients

We used a qualitative sampling plan to calculate the sample size with a specific focus on diversity of the participants based on the rule of thumb approach [22]. The goal was to obtain a heterogeneous sample. As previous studies show that the intensity of thirst can be influenced by various factors such as age, gender, fluid restriction and diuretic administration [14, 23], we considered these criteria. Other criteria included length of hospitalisation, duration of CHF and registration status for transplantation. Based on these criteria, we calculated a sample size of 10 interviews a priori. Participants were selected using purposive sampling to represent as many different patient situations as possible [24].

Inclusion criteria were experience with thirst in the context of CHF, listed for heart transplantation, ≥ 18 years, inpatient, sufficient knowledge of the German language and written informed consent. Exclusion criteria were an implanted VAD and/or cognitive impairment.

#### 2.2.2 Focus Groups and Survey With Nurses

We used FG to gather the experiences and opinions of nurses on the topic of thirst in patients with advanced CHF and planned two FG with 6–8 nurses each. For the survey, we recruited participants directly in hospitals with heart transplantations. We contacted 20 hospitals by email. Nine hospitals confirmed their participation and forwarded the e‐mail to nurses. Furthermore, we approached six organisations and professional nursing networks via e‐mail (e.g., German society for internal intensive care and emergency medicine or federal working group of nursing experts for persons with heart failure). For FG and survey, inclusion criteria were nurses who had completed at least 3 years of training in nursing (registered nurses), experienced in the care of adult patients during their waiting time for a heart transplantation and written informed consent.

### 2.3 Data Collection

#### 2.3.1 Interviews With Patients

We recruited the patients with support of the ward management and conducted the interviews on the ICU, IMC or general ward in separate rooms without disturbance or by telephone. All interviews were performed by an experienced female researcher with a nursing background (FW). The researcher is not involved in direct patient care as a nurse and therefore has no direct relationship with the study participants. Based on the research question and existing evidence, we prepared a pretested interview guide focused on experiences with thirst, needs and requirements, and experiences with information provided by healthcare professionals (Table S2). We also recorded socio‐demographic and disease‐related data using a short questionnaire (Table 1).

**TABLE 1:** Characteristics of patients in the interviews (*n* = 10).

Variable	*n* (%)
Age (years)	43.5 (± 15)^a^
Gender (female)	3 (30)
Duration of HF	25 days–21 years^b^
Fluid restriction	10 (100)
Amount of fluid	1.5–2 Litre per day^b^
Diuretics in total	9 (90)
Intravenous administration of diuretics	3 (30)
High urgency list	5 (50)
Duration of hospitalisation	7–116 days^b^
Re‐transplantation	1 (10)

Abbreviation: HF, heart failure.

^a^ Mean (standard deviation).

^b^ Range.

#### 2.3.2 Focus Groups With Nurses

Our pretested interview guide and the short questionnaire before the FG were developed based on the research question and existing evidence. FGs with nurses were performed in a meeting room in the hospital. Ward manager selected the participants by direct approach. We conducted the FG with two researchers who are not involved in direct patient care as nurses and therefore had no direct connection to the nurses on the ward. One researcher moderated the FG (FW), while the other logged important statements or side conversations (*SC*). At the beginning, the participants received a short questionnaire with seven items (Table S3a). Contents of the questionnaire and FG included the relevance and significance of thirst in patients with CHF and nursing care, causes of thirst, thirst detection and procedures for patient information and counselling (Table S3b).

#### 2.3.3 Survey With Nurses

Building on the findings of the FG and an earlier study [15], we developed an online survey using SoSci survey [25]. Prior to data collection, the survey was checked in a standardized pre‐test by seven nurses. The questionnaire contains a total of 20 items and, besides socio‐demographic data, focused on the relevance of thirst for patients and nursing care, causes of thirst, assessment of thirst, interventions to alleviate thirst, information sharing and advice provided by nurses (Table S4).

### 2.4 Data Analysis

#### 2.4.1 Qualitative and Quantitative Data

Qualitative data of the interviews and FG were digitally recorded and transcribed verbatim. We used MAXQDA [26] for data analysis and conducted a structured content analysis by Kuckartz [27]. Initially, at least two researchers (FW, LK, SC) developed the categories inductively from the transcripts. First, main categories were developed and the data were coded. The categories were then compared with each other, discussed with the experienced qualitative research team and harmonised with the topics of the interview guide (deductive approach). Afterwards, we formed subcategories based on the coded main categories. If necessary, the researchers were supported by a senior researcher (SK). Quantitative data of the survey and the FG questionnaire were analysed descriptively using IBM SPSS software (version 29).

#### 2.4.2 Mixed Methods

For the mixed methods analysis in general, we used a convergent design with one exception: we used a sequential exploratory approach to create the nurses' survey applying the results of the FG [28, 29]. Firstly, we analysed individual data collections. Afterwards, we compared results of both methodological approaches and summarised qualitative and quantitative data as meta‐inferences [29]. We developed common categories from all results and presented results as joint display with a side‐by‐side design [28, 29].

### 2.5 Ethical Considerations

The ethics committees of the Medical Faculty of the Ruhr University Bochum, based in East Westphalia (#2022‐897; 29 April 2022), and the Medical Faculty of the Heinrich‐Heine University Düsseldorf (#2022‐1996; 16 August 2022) approved the study. Afterwards, the study was registered at the German Clinical Trials Register (DRKS00029184). All participants who participated in the interviews and FG were informed in writing and verbally and gave their written informed consent. Participants in the survey were informed by email and gave their informed consent at the beginning of the survey. Participation was voluntary at all times.

## 3 Results and Findings

Overall data collection period ran from October 2022 to November 2023. We conducted ten interviews with hospitalised patients with advanced CHF waiting for heart transplantation, two FG with respectively five nurses and an online survey with 59 nurses in Germany.

### 3.1 Qualitative Findings

Qualitative findings of the interviews with patients and the FG with nurses are summarised in Figure 1 and will be reported as categories. Detailed presentation of the quotes is presented in Table S5.

**FIGURE 1:**
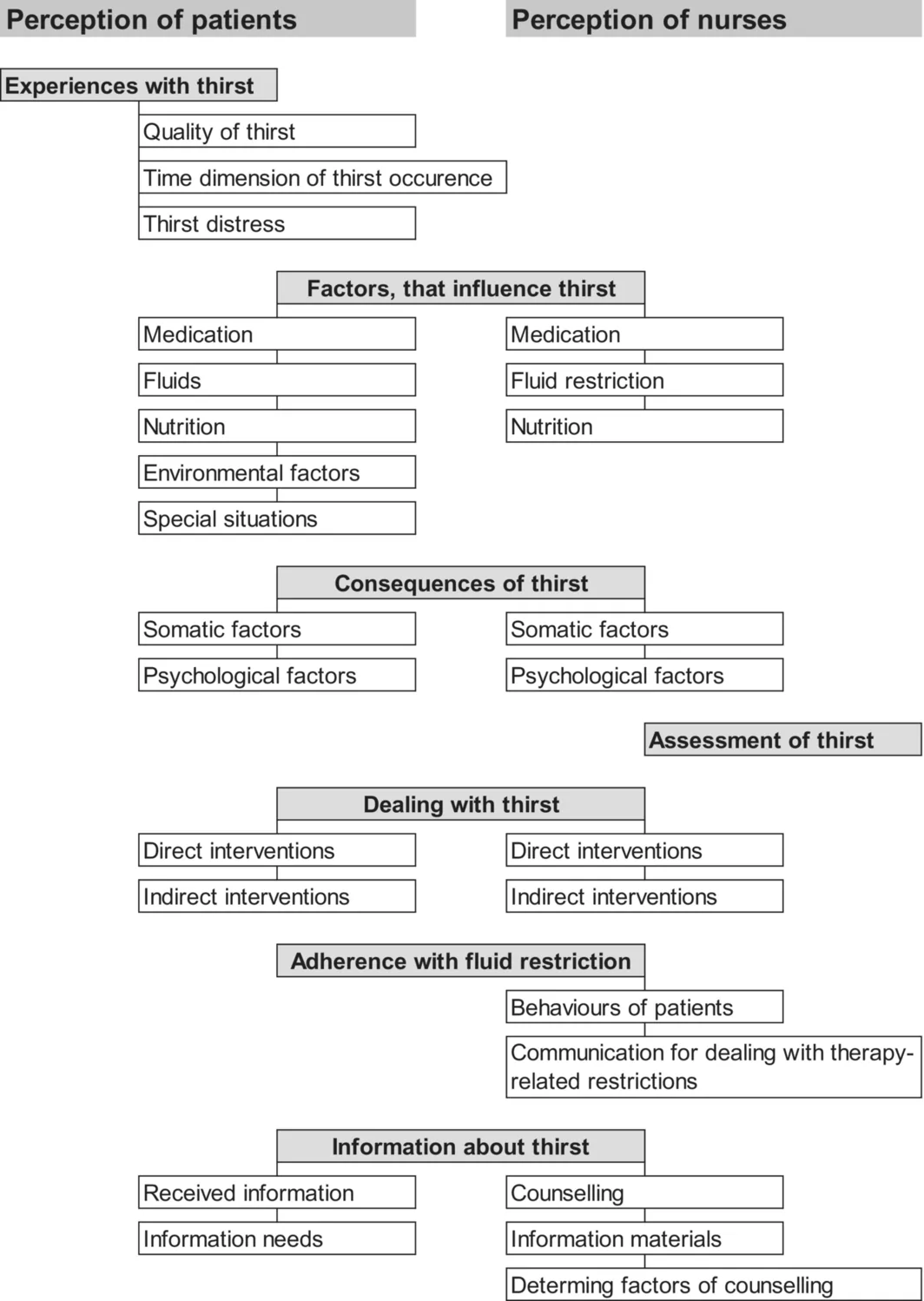
Main and subcategories from the interviews with patients and focus groups with nurses.

#### 3.1.1 Interviews With Patients

We conducted 10 interviews with hospitalised patients with advanced CHF (nine interviews on the ward, one interview by telephone) from November 2022 to November 2023. The average age of the patients was 43.5 (±15) years, three of them were female, and all had a prescribed fluid restriction (Table 1). The interviews lasted between 6 and 24 min. We identified six main categories and 14 subcategories.

##### 3.1.1.1 Experiences With Thirst

Patients experienced thirst differently. Some patients only felt a slight sensation of thirst, and others described their thirst as very strong. We divided the experience of thirst into three dimensions: quality of thirst, time dimension of thirst occurrence and thirst distress.

Patients described their feeling of thirst primarily as a dry oral feeling or having a strange taste. Others described their thirst as a strong desire to drink fluids. The time to perceive thirst is very different. For some patients, thirst was present throughout the day. Others felt thirst mostly in the morning, in the evening, or at night. Some described feeling particularly thirstier after eating or after taking medications, and for many patients, thirst intensified during hospitalisation. Some patients described feeling constricted and stressed due to thirst and a prescribed fluid restriction.

When you're thirsty and you know you're not allowed to drink. It drives you crazy. It increases the feeling of thirst immeasurably. (I2)

##### 3.1.1.2 Factors That Influence Thirst

Patients identified several influencing factors of thirst. We divided these factors into five subcategories: medication, fluid, nutrition, environmental factors and special situations. Most patients reported medication in general as an influencing factor; some patients stated that specifically diuretics influenced their thirst.

This roughly coincides with the start of taking this medication (diuretics) and the slightly increased feeling of thirst. (I4)

The criterion ‘fluids’ include fluid restriction, fever and oedema, which were described as having an impact on thirst. For the factor nutrition, in particular, salty food, sweets or spicy and garlic‐containing dishes were mentioned. Environmental factors include exercise, warm weather or air conditioning and dry room air.

##### 3.1.1.3 Consequences of Thirst

Patients experienced other symptoms due to their thirst. These can be divided into somatic and psychological factors. Somatic factors include dry mouth, changes in taste or dizziness and headaches. Some patients described a high level of suffering, stress and thought loops all day due to the feeling of thirst.

Thirst is terrible. Better to be hungry. (I2)

Furthermore, patients often associated their thirst with their prescribed fluid restriction, keeping them occupied throughout the day.

##### 3.1.1.4 Dealing With Thirst

Patients described interventions they have tried themselves. Many patients developed their own strategies for relieving thirst and tested several interventions. Overall, patients' responses vary greatly and are highly individual. We differentiated responses into direct and indirect interventions.

Direct interventions can reduce the dry oral feeling or the desire for fluids. These include fluid intake, cooling, flavours, carbon dioxide, moistening of the mouth, brushing teeth and division of fluid. As cooling interventions, patients named cold water, water ice or ice cubes and chemical colds such as menthol candies, with the taste perceived differently. Some find the use of different flavours helpful, whereas others find the taste of, for example, sweet mouth sprays rather thirst‐inducing.

And a mouth spray would certainly help too. It's just too sweet for me personally. (I8)

When dividing up the liquid, patients advised not to use large glasses, in order to drink more frequently over the hole day. Others monitor their daily fluid intake, for example, using an app.

Indirect interventions cannot directly reduce the feeling of thirst but can support direct interventions, for example, distraction, changes in environmental factors (e.g., fresh air supply) or lip care.

##### 3.1.1.5 Adherence With Fluid Restriction

All patients had a prescribed fluid restriction and stated to mostly adhere to it as they considered it important for them due to their advanced CHF, although some patients still sometimes drink more than the prescribed fluid per day due to intense thirst.

That you don't exaggerate, but are simply honest with yourself. That a single mistake or transgression can have a greater impact, especially at an advanced stage. (I1)

##### 3.1.1.6 Information About Thirst

This category can be divided into two subcategories: received information and information needs. Patients reported that they did not receive any information about thirst, but often received it on request. Most patients stated that they were only informed about the prescribed fluid restriction by their physicians or that this was stated in their medical reports.

Just about the amount you drink. That I shouldn't drink so much water. (I8)

Some patients required general knowledge about thirst, prompt solutions for thirst management and information flyers. Others felt no need for information, feeling sufficiently informed.

#### 3.1.2 Focus Groups With Nurses

We conducted two FG, each with five nurses from two different university hospitals in October and November 2022. The FG lasted 28 and 63 min. Socio‐demographic data are listed in Table 2. Before the FG began, the participants completed a short questionnaire. Results are listed below and in Table 3. We identified six main categories and 12 subcategories.

**TABLE 2:** Characteristics of nurses.

Variable	% (*n*)
Focus groups (*n* = 10)	
Working on a cardiac surgery unit/ward	100 (10)
Professional experience ≥ 5 years on the ward	50 (5)
Further training (e.g., mentoring, intensive care or management)	4 (40)
Academic degree	20 (2)
Survey (*n* = 59)	
Working in a hospital	81.4 (48)
Professional experience > 5 years in this field	54.2 (32)
Further training (e.g., intensive care, heart failure, transplantation or counselling)	67.8 (40)
Academic degree	28.8 (17)

**TABLE 3:** Joint display of qualitative and quantitative results.

Categories	Qualitative methods		Quantitative methods	
	Interviews with patients (*n* = 10)	Focus groups with nurses (*n* = 10)	Questionnaire before focus group (*n* = 10)	Survey with nurses (*n* = 59)
Experiences with thirst	Experience of thirst is very different. Some patients feel less thirsty, others feel very thirsty. Patients describe their thirst mainly as a dry feeling in the mouth, a change in taste and the desire to drink fluids. For some patients, thirst is present throughout the day. Others feel thirst mostly in the morning, in the evening or at night. Thirst can also increase after meals or taking medication. In addition, the feeling of thirst has changed for some during the hospital stay. Some patients describe that they feel constricted and stressed due to thirst. Patients reported somatic and psychological factors. Somatic factors were dry mouth, changes in taste or dizziness and headaches. Psychological factors were a high level of suffering and stress, thought loops and the strong thought of reduced fluid intake throughout the day.	Nurses reported that patients describe somatic factors such as dry mouth, a foggy feeling and low blood pressure as consequences of their thirst. Furthermore, they reported psychological factors such as circling thoughts, extreme burden and suffering, the feeling of loss of control or that the patients are unable to cope well with the situation.	Nurses rated the importance of thirst for patients on a scale from 0 to 10 (not important to very important) with a median of 7 (IQR 5.25–8). Relevance for nursing care was rated with a median of 7 (IQR 5.25–7.75).	Nurses (*n* = 56) rated the importance of thirst for patients on a scale from 0 to 100 (not important to very important) with a median of 76 (IQR 61–86). Relevance for nursing care was rated with a median of 71 (IQR 51–83; *n* = 57).
			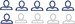 Six of 10 nurses stated that patients regularly reported thirst during their inpatient stay.	
			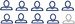 Nine nurses indicated that the patients’ feeling of thirst changed during their hospitality.	
Meta‐inferences	**Confirmed:** With a median of 7 (0–10) and 76/71 (0–100), nurses consider thirst to be a relevant issue for both patients and nursing care. Patients and nurses state that thirst levels change during hospital stay. **Expanded:** Patients’ experiences of thirst range from mild thirst to severe thirst. Both, patients (self‐assessment) and nurses (external assessment) reported somatic and psychological symptoms and signs that can occur due to increased thirst.			
Factors that influence thirst	Five categories were identified: medication (e.g., diuretics), fluids (fluid restriction, fever or oedema) nutrition (salty, sweet, spicy or garlic), environmental factors (exercise, weather, air conditioning or dry air) and special situations, for example, sedation due to an intensive intervention.	Nurses reported three factors that influence thirst in their perception: fluid restriction, nutrition and medication.	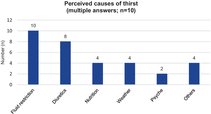	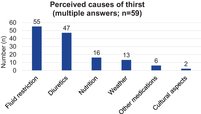
Meta‐inferences	**Confirmed**: There is a coherence between the patients’ and nurses’ statements regarding the factors medication, fluid intake and nutrition, which have a negative impact on thirst. **Expanded:** Patients cited additional factors that negative influence their thirst (environmental factors, e.g., exercise, weather, air conditioning or dry air and special situations, e.g., sedation due to an intensive intervention).			
Assessment of thirst	n.a.	Thirst is not recorded in a standardized way, but only individually or if symptoms of decompensation are present.	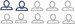 Two of 10 nurses (20%) stated that they regularly ask patients about thirst. 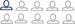 One person noted that there is a standardized procedure for recording thirst on the ward.	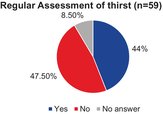 3.4% (*n*=2) report having a standardized procedure for measuring thirst. 28.8% (*n*=17) check thirst of patients daily or several times a day.
Meta‐inferences	**Confirmed:** The assessment of thirst was mentioned in all forms of data collection with nurses. **Discordant:** There is considerable heterogeneity in the measurement of thirst. 20%–44% report to assess thirst regularly. Only two nurses in the survey stated that they had a standardized procedure for assessment. In the qualitative evaluation, nurses report that they assess thirst irregularly and often only when the symptom occurs. One nurse in the quantitative survey prior to the focus group reported having a standardized procedure for recording thirst, but this is not reflected in the qualitative statements. None of the patients mentioned that they had been actively asked about their thirst by healthcare professionals.			
Dealing with thirst	Patients used direct interventions which reduce the dry feeling in the mouth or the desire for fluids: fluid intake; cooling (water ice, cold water, cooling sprays, chemical cold, e.g., menthol), refreshing flavours, for example, in form of candies or toothpaste, carbon dioxide, division of fluids, moistening of the mouth. They also use indirect interventions that support the direct measures (e.g., lip care, air supply or distraction).	Nurses reported direct intervention, which they recommend patients to reduce their thirst: cooling, flavours and moistening the mouth. As indirect interventions they prefer advise to cream their lips and skin. They also advice nutritional aspects and to use small glasses or keep the bottles out of reach.	n.a.	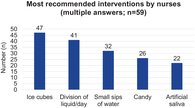
Meta‐inferences	**Confirmed:** Patients and nurses both mentioned ice cubes, water ice, rinsing the mouth, using small glasses and lip care as interventions to reduce thirst. **Expanded:** Patients reported that different flavours help in context of cold water, water ice or ice cubes and chemical colds like menthol candies to reduce their thirst. **Discordant:** Patients and nurses also mentioned different interventions to reduce thirst. Patients named additionally cold water and carbon dioxide, menthol candies, dividing the liquid and using an app, distraction, fresh air and brushing teeth. Nurses also recommended lemon sticks, cucumber or watermelon, reducing salt and sugar intake and skin care.			
Information about thirst	Patients reported that they did not receive any information about the feeling of thirst, but often received it upon request. Most patients were informed about adherence to fluid restriction. They stated that they mostly adhere to fluid restriction and that it is important for them due to their advanced CHF. Some patients told to drink sometimes more. Some patients wanted general knowledge about thirst, solutions to reduce thirst and information flyers. Others had no need for information and felt that they were sufficiently informed.	Nurses report a lack of a unified, standardised counselling concept, emphasizing the need for consistent information across the ward while also valuing individualised approaches. They find visual aids like flyers, booklets and images helpful for advising patients on managing thirst and emphasize the importance of carefully selecting appropriate images for these materials. They identify personal factors (like knowledge about thirst), ward‐related factors (such as environment, time, materials, training and documentation), and patient‐related factors (including disease understanding and adherence) as influencing successful information transfer. Nurses describe non‐adherent patient behaviours as stemming from intransigence, denial of illness or anxiety. Some patients appear to be in psychological denial about their chronic condition, leading to defensive or avoidant behaviours that hinder adherence to treatment. Nurses primarily focus on fluid restriction adherence, using education and worst‐case scenarios to convey its importance. They emphasize the need for individualised approaches but note that some patients seem resistant to their advice.	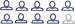 Nine of 10 nurses (90%) mentioned that they **do not have** a structured procedure on the ward for counselling relating to thirst.	**Information about thirst** The importance of informing patients about thirst was indicated on a scale of 0‐100 (not important to very important) with a median of 81 (IQR 56‐100; *n* = 53). In total, 8.5% (*n* = 5) stated to hand out information material on thirst. Of these, four nurses (80%) reported that the materials was evidence‐based. **Counselling of patients** 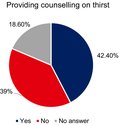 Topics of the counselling included, for example, general information about the disease, development and causes of thirst, intervention of thirst reduction, fluid restriction and coping strategies. Nurses rated the importance of developing a counselling intervention (0 = not important to 100 = very important) with a median of 74.5 (IQR 51.75–94.25; *n*=50). As conditions for counselling, 83.1% (*n* = 49) cited a quiet environment; 71.2% (*n* = 42) mentioned the involvement of relatives, and 22% (*n* = 13) listed other factors such as time resources, visual aids and patient‐related factors (adherence, understanding of their illness).
Meta‐inferences	**Confirmed**: Nurses stated a standardized information for a counselling as useful. Nurses and patients find it useful to have visual aids like flyers to receive information in context of thirst and thirst management in CHF. Nurses reported the importance of individual counselling, which is also desired by patients. **Expanded:** Nurses reported about a missing counselling concept and different factors of both themselves and patients that can influence counselling, like knowledge or spatial environment. **Discordant:** The importance of informing patients about thirst was rated high by nurses, while patients sometimes had no need for information. Patients and nurses provide partly conflicting accounts of adherence to fluid restriction. While patients generally describe themselves as adherent and downplay occasional deviations, nurses tend to attribute non‐adherence to denial, resistance or anxiety.			

**Abbreviations:** CHF, chronic heart failure; IQR, interquartile range.

##### 3.1.2.1 Factors That Influence Thirst

Nurses described various factors which influence thirst, including fluid restriction, nutrition and medication. They perceived that fluid restriction leads to increased stress for patients and that their thirst increases accordingly. Nurses also had the feeling that many patients drank more before their illness and the prescribed amount was not enough to quench their thirst.

As soon as […] their intake is reduced to a maximum of 1.5 litres, this psychological pressure is then present. (FG 2)

Nutrition is a further factor influencing thirst and the nurses perceived that salty, sweet and spicy foods particularly promote thirst. They also explained that food is very different in other cultures and many patients are provided with strongly flavoured food by relatives. Nurses also experienced that some medications promote thirst, for example diuretics and catecholamines.

##### 3.1.2.2 Consequences of Thirst

Nurses described psychological and somatic factors that can arise due to thirst. Somatic factors included dry mouth, a foggy feeling and low blood pressure. Psychological factors included circling thoughts, extreme burden, the feeling of loss of control or that patients are unable to cope well with the situation.

There is often no other topic. Then it's all about that. (FG 1)

##### 3.1.2.3 Assessment of Thirst

Nurses reported that thirst is not assessed in a standardised way on the ward and they do not use a scale. Thirst is often recorded when patients actively mention it. If symptoms and signs of decompensation occur, nurses ask whether the patients have been drinking more.

We all do it individually in our daily routine, when we realise that there is a problem. (FG 2)

##### 3.1.2.4 Dealing With Thirst

Nurses named direct and indirect thirst‐relieving interventions they suggest to patients. As direct interventions, nurses recommend cooling interventions such as ice cubes or water ice, flavours such as lemon sticks, moistening the mouth and foods such as cucumber or watermelon.

Indirect interventions mentioned by the nurses include moisturising or applying cream to the lips and the skin. Furthermore, they advise their patients to reduce salt and sugar, use small glasses and keep water bottles out of reach.

[…] pour yourself small glasses, maybe put the bottle on the windowsill, teach your‐self a bit of self‐discipline, […]. (FG1)

##### 3.1.2.5 Adherence With Fluid Restriction

This category can be divided into behaviours of patients and communication for dealing with therapy‐related restrictions. Nurses described non‐adherent behaviours by explaining that some patients are intransigent, do not want to acknowledge their illness or are anxious. Examples of unresponsiveness to therapy‐related behaviour are ordering salty food or drinking a bottle of water. Others described that some patients are in a phase in which they do not want to recognise the condition of their chronic illness and are therefore non‐adherent.

The patients are ill and waiting for a heart. And this is an exceptional situation for them […]. They might go through a phase like that, they don't want to realise it. (FG1)

Nurses stated, that they advise about adherence to fluid restriction and possible consequences of non‐adherence by either applying educational methods or demonstrating worst‐case scenarios. They found it important to consider each patient individually. Some nurses reported that some patients are resistant to advice.

##### 3.1.2.6 Information About Thirst

Information on thirst provided by nurses can be categorised into three themes: counselling, information materials and determining factors of counselling.

Nurses stated that they do not currently have a unified counselling concept with a clear common thread. They consider standardised information for counselling by nurses important but also prefer a high degree of individuality within the counselling.

[…] we don't have a standardised concept where we somehow have a path right from the start. (FG 1)

Nurses reported that printed and visual aids such as specific flyers or booklets and images for advice on thirst would be helpful and found that choosing suitable pictures for informational materials was very important.

They also listed factors that may influence counselling: personal factors, for example, specific knowledge on the topic of thirst, ward‐related factors, for example, a spatial environment, time resources, available materials, training courses, handover and documentation structures. They also reported patient‐related factors, for example, general knowledge about the disease and adherence.

### 3.2 Quantitative Results

Quantitative results are briefly presented below. Characteristics of participants are listed in Table 2. Further results are presented in Table 3.

#### 3.2.1 Questionnaire Before Focus Group

All nurses who participated in the FG (*n* = 10) completed a short questionnaire. They rated the importance of patients' thirst with a median score of 7 (IQR 5.25–8) on a numerical rating scale from 0 to 10, with 10 indicating very important. Relevance for nursing care was rated with a median of 7 (IQR 5.25–7.75). Six nurses reported that patients often mention thirst during their hospitalisation, while nine nurses noted changes in patients' thirst during their hospital stay. Two nurses stated they routinely ask patients about thirst, and one nurse mentioned a standardised procedure for the assessment of thirst on the ward.

#### 3.2.2 Survey With Nurses

The survey was conducted between February and July 2023 with 59 nurses participating. On a visual analogue scale from 0 to 100 (0 = not important, 100 = very important), nurses assessed the importance of thirst for patients with a median score of 76 (IQR 61–86; Min = 12, Max = 100; *n* = 56). The perceived relevance of thirst in nursing care was rated with a median score of 71 (IQR 51–83; min = 21, max = 100; *n* = 57). In total, 26 (44.1%) nurses reported to routinely assess patients' thirst, 17 (28.8%) assess thirst once to several times a day, and 2 (3.4%) stated to have a standardised procedure for assessing thirst in their workplace. In total, 53 nurses (89.8%) recommend thirst‐relieving interventions. The most common intervention was ice cubes (79.7%; *n* = 47). The importance of informing patients about thirst was rated with a median score of 81 (IQR 56–100; min = 31, max = 100; *n* = 53).

### 3.3 Integration of Findings

We combined qualitative and quantitative results, created a joint display (Table 3) and described meta‐inferences. We constituted five common categories: experiences of thirst, factors that influence thirst, assessment of thirst, dealing with thirst and information about thirst. The quantitative data show the relevance of thirst as a symptom for patients. The experiences of patients and nurses recorded in the qualitative data confirm these results and additionally highlight the impact of thirst.

## 4 Discussion

This study aimed to assess the experiences and needs of patients and nurses regarding thirst in patients with advanced CHF waiting for heart transplantation on ICU, IMC or general ward. Five common categories were identified in the meta‐inferences. Overall, patients perceived their thirst individually and used specific personal strategies to alleviate it. Nurses described high relevance for this topic in nursing care. There is a lack of uniform concepts on the wards for assessing thirst, information provision and symptom relief concepts.

Thirst is perceived differently by patients and was sometimes described as very distressing. This is in line with the study results by Zheng et al. [30] who describe that patients experience a high level of discomfort due to thirst. This has also been shown in quantitative studies, were up to 73% of participants reported moderate to severe thirst distress [8, 9, 31]. Our results and those of Zheng et al. [30] show that the feeling of thirst has physical (e.g., dizziness) and psychological (e.g., circling thoughts) characteristics. This should be considered when assessing symptoms and selecting interventions. Raising awareness in healthcare practice is therefore important, as only about half of the nurses in our study stated that they regularly assess thirst. The use of validated instruments such as the thirst distress scale for patients with heart failure or a visual analogue scale to measure thirst intensity could therefore be helpful [32].

Although several potential causes of thirst were described by both participant groups in both qualitative and quantitative data, fluid restriction still appears to be the most relevant factor that intensifies the feeling of thirst. Although guidelines recommend fluid restriction in advanced CHF [4], surprisingly there is a lack of evidence on the impact of fluid restriction in advanced CHF. Herrmann et al. [33] investigated the effect of liberal fluid intake versus strict fluid restriction in patients with stable CHF, showing no advantage for either strategy in terms of participants' health status. Further studies investigating liberal fluid intake in patients with advanced CHF are necessary. Results can be used to plan adequate patient counselling and self‐management strategies based on patients' needs and preferences in the context of shared decision‐making approaches.

In order to be able to develop suitable interventions, it is important to consider the perspectives of patients [20]. Our results show that nurses mentioned similar interventions to those reported by patients, but patients listed further interventions and reported using several interventions to reduce thirst. In their review, Pico‐Munyoz et al. [34] describe that applying various oral stimuli, such as ice, carbon dioxide and flavours, is seen as most helpful to reducing thirst. Overall, results show that individually tailored interventions appear to be beneficial for patients. However, previous studies have predominantly examined single interventions [7, 11, 12, 35] which seem not adequate considering the results of this study. Only one Chinese study describes the development and feasibility of a complex intervention regarding thirst relief in patients with CHF using especially traditional Chinese medicine interventions such as acupressure [13], which may not be culturally applicable in many countries, and requires profound knowledge and training by experts to be universally applicable, which shows that interventions should be adapted to cultural needs.

Although, nurses stated that counselling is important, it is rarely provided. Consequently, many patients reported that they had not received any information from healthcare professionals about thirst. The inability to share information may be related to the factors such as insufficient knowledge about thirst or lack of time. The need for counselling and knowledge provision was also mentioned in the study by Zheng et al. [30]. Profound knowledge about thirst in patients with CHF including knowledge on assessment of thirst, causes of thirst and suitable interventions are important for nurses, but there is limited evidence on how nurses acquire information and which information they provide to patients. There is only one study in which healthcare professionals were asked about possible measures to reduce thirst in patients with CHF [15]. In addition, Zheng et al. [13] interviewed nurses about current thirst management strategies as part of their intervention development. A cross‐sectional study in which nurses in surgical ICUs were asked about their knowledge about thirst management showed that only few had received training and that there was an obvious lack of knowledge [36]. These results demonstrate the relevance for training programs.

The results of our study are important for the development of thirst management interventions, showing that the intervention should consider patients' knowledge, thirst‐inducing factors and strategies for thirst reduction. As many factors influence thirst [7], it is important to determine which factors play key roles in the patient's individual situation. Information materials for both, patients and nurses, are also necessary and should be adapted to the situation and setting. The quantitative data identified factors that are relevant for education, such as patient‐related factors like adherence and knowledge. The qualitative data confirm these findings but also provide more detailed practice examples.

Based on these results, we are planning to develop a counselling intervention on thirst for patients with advanced CHF, including feasibility testing, nurse training and a pilot evaluation within a before‐and‐after design and process evaluation [19].

### 4.1 Strengths and Limitations

Our study has several strengths. We used a mixed methods design, included various perspectives and analysed qualitative and quantitative data together. The importance of mixed methods is illustrated, for example, in the integration of nurses' statements regarding the relevance of thirst in practice in the survey, alongside the more in‐depth experiences of patients and nurses gathered through interviews and FG. The categories of the qualitative data were developed by two researchers, regularly supported by a senior researcher and discussed within the team.

Our study has also limitations. Due to their state of health, some of the interviews with patients were very short. Nevertheless, we were able to include a heterogeneous collective of patients and important information was gathered in the majority of cases. The questionnaire was not formally validated, but pretested. The sample size of the survey with nurses was relatively small, even though we contacted many hospitals and professional nursing networks. However, we specifically consulted nursing experts in the field of CHF. Furthermore, due to shift work and time constraints, we were only able to include five nurses in each FG. Given the descriptive research question and the expertise of the included nurses, the sample appears adequate to draw conclusions on the research questions and as a basis for the development of new interventions.

### 4.2 Implications for Practice and Further Research

Our results show that both patients and nurses demonstrate a need for professional advice regarding thirst in the context of advanced CHF. It is important to apply consistent procedures on the wards and still tailor interventions to individual patients. Currently, there is a lack of evidence on the effects of such complex interventions, which is strongly needed considering the relevance of thirst for patients with advanced CHF.

## 5 Conclusion

Thirst in hospitalised patients with advanced CHF waiting for transplantation can be very pronounced and is perceived in different ways by patients. A uniform procedure for assessing and alleviating thirst on the ward is important. Involvement of patients and nurses is useful for developing a counselling intervention.

## Funding

The study was financed by own funds of the Heart and Diabetes Center NRW, Ruhr University Bochum and Institute of Nursing Science, University of Cologne, Faculty of Medicine and University Hospital Cologne.

## Ethics Statement

The ethics committee of the Medical Faculty of the Ruhr University Bochum, based in East Westphalia (#2022‐897; 29 April 2022), and the Medical Faculty of the Heinrich‐Heine University Düsseldorf (#2022‐1996; 16 August 2022) have given a favourable ethics vote.

## Consent

Before the beginning of the study, the participants signed an informed written consent form.

## Conflicts of Interest

The authors declare no conflicts of interest.

## Supporting information


**Table S1:** Good reporting of a mixed methods study (GRAMMS) checklist.

**Table S2:** Interview guide patients.

**Table S3:** Questionnaire and interview guide of the focus groups with nurses.

**Table S4:** Survey with nurses.

**Table S5:** Quotes of the interviews with patients and focus groups with nurses.

## Data Availability

Data are available by the first author under consideration of aspects of protection of data of the participants.
