# Supplementary material for: Thirst in Patients With Advanced Chronic Heart Failure Waiting for a Heart Transplantation: A Mixed Methods Study of Patients' and Nurses' Experiences
Source: Nurs Crit Care. 2026 Oct 7;31(6):e70686. doi: 10.1111/nicc.70686 (PMC13643929; doi:10.1111/nicc.70686)
Supplement: Supplementary file 1 [file NICC-31-0-s002.docx]

**Table S2. Interview guide patients**

| **Introduction:**  It is known that patients with chronic heart failure often suffer from increased thirst. I would therefore like to know how you perceive this symptom. | |
| --- | --- |
| **Experience with the feeling of thirst** | |
| 1. What experiences do you have with thirst in the context of your chronic heart failure? | - How would you describe your thirst? (Dimensions of symptoms: quality, intensity, distress, time dimension)   - How does your thirst feel?   - How often do you feel thirsty?   - Are there times of day when you feel thirstier?   - Do you feel that your thirst has changed during your stay?   - Do you feel burdened because of your thirst? - What do you think triggers your thirst? - In your opinion, what factors influence your thirst? |
| **Needs and requirements** | |
| 2. Can you please describe your needs in relation to your thirst? 3. What interventions help you to reduce thirst? | - What are your needs when you think about your thirst? |
| **Experiences with providing information and advice to healthcare professionals** | |
| 4. What experiences do you have with the information and advice provided by healthcare professionals (e.g. physicians or nurses) regarding thirst as a symptom of your chronic heart failure? | - How did you receive the information? - In what context did the consultation take place? - Who advised you? (e.g. hospital, family doctor, outpatient clinic) - What interventions were recommended to you? - Are there differences in the advice given by different healthcare professionals, e.g. nursing staff, medical staff, etc.? If so, please describe them. - Were there any moments when you felt you did not receive sufficient information or advice? When did you notice this? - What information would you have needed? |
| **Important topics relating to thirst** | |
| 5. Which topics do you consider important with regard to your thirst? | |
| **Tips for other affected persons** | |
| 6. What advice would you give to other patients with chronic heart failure, apart from drinking fluids to reduce thirst? | |
| **Conclusion** | |
| 7. Is there anything else on the subject of thirst or our study that has not yet been addressed? | |
| **Note from interviewer**  **Situation of the interview**  Overall impression:  Spatiality:  Faults/interruptions:  **Narrative flow:**  Atmospheric impression:  Emotions during the interview:  **Conversation topics**  Was not addressed/avoided: | **End of conversation**  Questions after the interview with the patient:  Mood at the farewell:  Other comments: |
