# Supplementary material for: Thirst in Patients With Advanced Chronic Heart Failure Waiting for a Heart Transplantation: A Mixed Methods Study of Patients' and Nurses' Experiences
Source: Nurs Crit Care. 2026 Oct 7;31(6):e70686. doi: 10.1111/nicc.70686 (PMC13643929; doi:10.1111/nicc.70686)
Supplement: Supplementary file 2 [file NICC-31-0-s003.docx]

**S3a.** Questionnaire before focus groups

| **Item** | **Response category** |
| --- | --- |
| 1. In your experience, how important is thirst for patients with chronic heart failure who are on the waiting list for a heart transplantation? | Numeric rating scale from 0-10 (not important to very important) |
| 2. In your opinion, how relevant is increased thirst in nursing care? | Numeric rating scale from 0-10 (not relevant to very relevant) |
| 3. In your opinion, what are the common causes of prolonged thirst among these patients (multiple answers possible)? | Fluid restriction, diuretics, other drugs (open answer), nutrition, cultural aspects (open question), weather, others (open question) |
| 4. Do these patients regularly express thirst during their hospital stay? | Yes, no |
| 5. Do you regularly ask these patients about their thirst?  If so, is there a standardized procedure on your ward? | Yes, no  Yes, no |
| 6. Do you feel that these patients' thirst levels change during their hospital stay?  If so, how does it change? | Yes, no  Open Answer option |
| 7. Is there a structured approach to advising these patients on this topic in your ward? | Yes, no |

**S3b.** Interview guide focus group

**Introduction:**

Patients with advanced chronic heart failure who are waiting for a heart transplantation may experience increased thirst due to their condition and therapeutic regiment. We would therefore like to explore your experiences and opinions on this topic.

**Experiences regarding patients' thirst**

Are you aware of situations in your ward where patients with chronic heart failure who are waiting for a heart transplantation experience thirst? Please describe these situations.

**Dealing with the symptom in nursing care**

How do you assess thirst of these patients on your ward?

How do you address this topic with patients?

**Information and counseling on thirst**

When informing or advising patients about the symptom, how do you proceed or how would you proceed (e.g. recommendations for interventions to relieve thirst)?

In your opinion, what factors influence the success of counseling in your ward? Please name both inhibiting and promoting factors.

What requirements need to be satisfied for nursing staff to be able to provide advice (e.g. general conditions, qualifications, skills, content of the advice)?

**Conclusion**

Are there any other aspects you would like to add on this topic?
