# Supplementary material for: Thirst in Patients With Advanced Chronic Heart Failure Waiting for a Heart Transplantation: A Mixed Methods Study of Patients' and Nurses' Experiences
Source: Nurs Crit Care. 2026 Oct 7;31(6):e70686. doi: 10.1111/nicc.70686 (PMC13643929; doi:10.1111/nicc.70686)
Supplement: Supplementary file 3 [file NICC-31-0-s004.docx]

**Table S1**. Good Reporting of A Mixed Methods Study (GRAMMS) Checklist

| **Guideline** | **Section: Page** |
| --- | --- |
| Justification to use a mixed methods approach to the research question. | Aims, page 2 |
| Articulation of the design in terms of purpose, priority, and sequence of methods. | 2.1., page 2 |
| Describe each method in terms of sampling, data collection and analysis. | 2. Methods, page 2-4 |
| Delineate where and how integration occurs and who has  participated in it. | 2.4.2., page 3 and 3.3, page 10, table3 joint display |
| Describe any limitation of one method associated with the presence of another. | 4.1, page 13-14 |
| Describe insights gained from mixing or integrating methods. | Table 3 joint display, 4. Discussion, page 10-11 |

Reference:

O'Cathain A, Murphy E, Nicholl J. The quality of mixed methods studies in health services research. J Health Serv Res Policy. 2008;13(2):92-8.
